# Supplementary material for: Extreme neonatal hyperbilirubinaemia in refugee and migrant populations: retrospective cohort
Source: BMJ Paediatr Open. 2020 May 28;4(1):e000641. doi: 10.1136/bmjpo-2020-000641 (PMC7264833; doi:10.1136/bmjpo-2020-000641)
Supplement: Author's manuscript [file bmjpo-2020-000641.draft_revisions.pdf]

BMJ Paediatrics Open

BMJ Paediatrics Open is committed to open peer review. As part of this commitment we make the peer review history of every article we publish publicly available.

When an article is published we post the peer reviewers' comments and the authors' responses online. We also post the versions of the paper that were used during peer review. These are the versions that the peer review comments apply to.

The versions of the paper that follow are the versions that were submitted during the peer review process. They are not the versions of record or the final published versions. They should not be cited or distributed as the published version of this manuscript.

BMJ Paediatrics Open is an open access journal and the full, final, typeset and author-corrected version of record of the manuscript is available on our site with no access controls, subscription charges or pay-per-view fees (http://bmjpaedsopen.bmj.com).

If you have any questions on BMJ Paediatrics Open's open peer review process please email info.bmjpo@bmj.com

BMJ Paediatrics Open

THE CONSEQUENCES OF EXTREME NEONATAL HYPERBILIRUBINEMIA IN A RESOURCE-LIMITED SETTING

Journal:	*BMJ Paediatrics Open*
Manuscript ID	bmjpo-2020-000641
Article Type:	Original research
Date Submitted by the Author:	12-Jan-2020

Complete List of Authors:	Wouda, Eva; Shoklo Malaria Research Unit; University of Groningen, University Medical Center<br>Thielemans, Laurence; Shoklo Malaria Research Unit; Universite Libre de Bruxelles, Neonatology-Pediatrics, Hôpital Erasme<br>Darakamon, Mue; Shoklo Malaria Research Unit<br>Nge, Aye; Shoklo Malaria Research Unit<br>Say, Wah; Shoklo Malaria Research Unit<br>Khing, Sanda; Shoklo Malaria Research Unit<br>Hanboonkunupakarn, Borimas; Mahidol Oxford Tropical Medicine Research Unit, Faculty of Tropical Medicine<br>Ngerseng, Thatsanun ; Mahidol Oxford Tropical Medicine Research Unit, Faculty of Tropical Medicine<br>Landier, Jordi; Shoklo Malaria Research Unit; Universite Aix Marseille, IRD, INSERM, SESSTIM<br>van Rheenen, Patrick; University Medical Center Groningen, Paediatric Gastroenterology<br>Turner, Claudia; Cambodia Oxford Medical Research Unit; University of Oxford Centre for Tropical Medicine and Global Health, Nuffield Department of Medicine<br>Nosten, Francois; Shoklo Malaria Research Unit; University of Oxford Centre for Tropical Medicine and Global Health, Nuffield Department of Medicine<br>McGready, Rose; Shoklo Malaria Research Unit; University of Oxford Centre for Tropical Medicine and Global Health, Nuffield Department of Medicine<br>carrara, verena; Shoklo Malaria Research Unit; Swiss Tropical and Public Health Institute, Department of Medicine

Keywords:	Jaundice, Neurodevelopment, Neurodisability

SCHOLARONE™
Manuscripts

I, the Submitting Author has the right to grant and does grant on behalf of all authors of the Work (as defined in the below author licence), an exclusive licence and/or a non-exclusive licence for contributions from authors who are: i) UK Crown employees; ii) where BMJ has agreed a CC-BY licence shall apply, and/or iii) in accordance with the terms applicable for US Federal Government officers or employees acting as part of their official duties; on a worldwide, perpetual, irrevocable, royalty-free basis to BMJ Publishing Group Ltd ("BMJ") its licensees and where the relevant Journal is co-owned by BMJ to the co-owners of the Journal, to publish the Work in this journal and any other BMJ products and to exploit all rights, as set out in our licence.

The Submitting Author accepts and understands that any supply made under these terms is made by BMJ to the Submitting Author unless you are acting as an employee on behalf of your employer or a postgraduate student of an affiliated institution which is paying any applicable article publishing charge ("APC") for Open Access articles. Where the Submitting Author wishes to make the Work available on an Open Access basis (and intends to pay the relevant APC), the terms of reuse of such Open Access shall be governed by a Creative Commons licence – details of these licences and which Creative Commons licence will apply to this Work are set out in our licence referred to above.

Other than as permitted in any relevant BMJ Author's Self Archiving Policies, I confirm this Work has not been accepted for publication elsewhere, is not being considered for publication elsewhere and does not duplicate material already published. I confirm all authors consent to publication of this Work and authorise the granting of this licence.

Confidential: For Review Only

THE CONSEQUENCES OF EXTREME NEONATAL HYPERBILIRUBINEMIA IN A RESOURCE-LIMITED SETTING

Eva M N Wouda[1,2], Laurence Thielemans[1,3], Mue C Darakamon[1], Aye A Nge[1], Wah Say[1], Sanda Khing[1], Borimas Hanboonkunupakarn[4], Thatsanun Ngerseng[4], Jordi Landier[1,5], Patrick F van Rheenen[2], Claudia Turner[6,7,8], Francois H Nosten[1,4,6], Rose McGready[1,4,6], Verena I Carrara[1,9]

[1]Shoklo Malaria Research Unit, Faculty of Tropical Medicine, Mahidol University, Mae Sot, Thailand;

[2]University of Groningen, University Medical Center Groningen, the Netherlands;

[3]Neonatology-Pediatrics, Cliniques Universitaires de Bruxelles - Hôpital Erasme, Université Libre de Bruxelles, Belgium;

[4]Mahidol-Oxford Tropical Medicine Research Unit (MORU), Faculty of Tropical Medicine, Mahidol University;

[5]Aix Marseille University, IRD, INSERM, SESSTIM, Marseille, France;

[6]Centre for Tropical Medicine and Global Health, Nuffield Department of Medicine, University of Oxford;

[7]Cambodia-Oxford Medical Research Unit, Angkor Hospital for Child, Siem Reap, Cambodia;

[8]Angkor Hospital for Children, Siem Reap, Cambodia;

[9]Department of Medicine, Swiss Tropical and Public Health Institute, Basel, Switzerland

Corresponding author: Verena I. Carrara, verena@shoklo-unit.com, +66 (0)55532026

Confidential: For Review Only

Keywords: neonatal hyperbilirubinemia, neonatal jaundice, extreme neonatal hyperbilirubinemia, kernicterus, neurological outcome, neurodevelopment, limited-resource setting, low- and middle-income countries, refugee, migrant

Confidential: For Review Only

Abstract

Objectives To describe neonatal survival and long-term neurological outcome in neonatal hyperbilirubinemia (NH) with extreme serum bilirubin (SBR) values.

Design Retrospective chart review; a one-off neurodevelopmental evaluation.

Setting Special Care Baby Unit at the Thailand-Myanmar border with phototherapy facilities, but limited access to exchange transfusion.

Patients Neonates ≥ 28 weeks of gestational age with extreme SBR values and/or acute neurological symptoms; neurodevelopment evaluation compared to that of children without any extreme SBR values or neurological symptoms, conducted at 36-83 months of age.

Main outcome measures Neonatal mortality rate; prevalence of acute bilirubin encephalopathy (ABE) signs; prevalence of delayed development scores based on the Griffiths Mental Development Scale (GMDS).

Results From 2009 to 2014, 1,946 neonates were diagnosed with jaundice; 129 (6.6%) had extreme SBR values during NH (extreme NH). In this group median peak SBR (inter-quartile-range (IQR)) was 430 (371-487) µmol/L and prevalence of ABE was 28.2%. Extreme NH-related mortality was 10.9% (14/129). Median percentile GMDS-general score of 37 survivors of extreme NH was three times lower than that of 39 children without extreme NH: 11 (2-42) *vs.* 31 (4-67), p=0.025. Four (10.8%) extreme NH survivors had normal development score (≥50th centile). Two (5.4%) had severe neurological impairment.

Conclusions Neonatal survival after extreme NH was high as were the deleterious consequences with poor neurodevelopmental outcomes. Early identification and adequate treatment of NH where exchange transfusion is not readily available is key to minimizing the risk of extreme SBR values or neurological symptoms.

Confidential: For Review Only

Background

In the most severe form of neonatal hyperbilirubinemia (NH), serum bilirubin (SBR) levels rise to extreme values causing a life-threatening condition that can lead to lifelong disability.[1] Acute Bilirubin Encephalopathy (ABE) happens during the early phase of the disease producing a vast array of neurological symptoms from mild and reversible to severe and irreversible. Its physiopathology is complex, however prematurity, sepsis, Glucose-6-phosphate dehydrogenase (G6PD) deficiency and ABO/ Rhesus incompatibility contribute to its development.[2-5] ABE may progress to kernicterus the most severe form;[6] subtler disabilities include diminished intelligence quotient, language delay, attention deficit/hyperactivity disorder and autism.[7]

A review of the worldwide burden of severe NH published in 2017 reported an incidence of 251.3 per 10,000 livebirths in South-East Asia the second highest after Africa.[8] NH-related mortality is high: 1,309 deaths per 100,000 live births in 2016.[9] Low- and middle-income countries bear the heaviest burden; two thirds of deaths and kernicterus cases occur in Sub-Saharan Africa and South Asia, 3 to 20% of survivors have normal long-term development.[10-13]

Most NH-related research follows the American Academy of Pediatrics guideline for neonates >35 weeks estimated gestational age (EGA) in which SBR levels >25 mg/dl (428 µmol/L) at age >72 hours are considered extreme and require exchange transfusion (ET).[14] The British National Institute for Health and Clinical Excellence (NICE) guideline proposes dynamic treatment thresholds based on postnatal age and varying with gestational ages from 23 to ≥38 weeks.[15] SBR values considered extreme therefore start as low as 13 mg/dl (230 µmol/L) for the youngest EGA. However publications reporting outcomes of neonates treated following these dynamic treatment thresholds are still rare.[16]

Confidential: For Review Only

The Shoklo Malaria Research Unit (SMRU) set up a Special Care Baby Unit (SCBU) in 2008 to provide basic care for neonates in Mae La, the most populous refugee camp along the Thailand-Myanmar border.[(17)] Starting 2011, another two SCBU's were set up in clinics for migrant populations. In livebirths from 2009-2014 NH prevalence was 18%; 9% of these neonates had at least one SBR value exceeding the NICE threshold at which ET should be considered.[(18)] ET is an intervention with potentially serious complications performed in an intensive care unit rather than a primary health facility.[(19, 20)] Due to the inherent difficulties of sending a refugee or migrant person to a specialised centre, only some neonates could be referred.

The objective of this review was to evaluate the immediate and long term clinical and neurodevelopmental outcomes of children from a low-resource setting with SBR values rising to levels considered extreme.

Methods

Three SMRU SCBU facilities contributed to the data; in refugees (1st January 2009–31st December 2014,[(17)]), and in migrants (1st January 2011–31st December 2014), **Figure 1.**

Inclusion criteria

Clinical charts of all liveborn singletons ≥28 weeks EGA without major congenital abnormalities admitted for a clinical diagnosis of jaundice were reviewed.

Definitions

For this analysis, NH with SBR values rising above levels that could justify ET, reported in the text as 'extreme NH' was defined as either i) two consecutive SBR measurements above the ET threshold of the NICE guideline; ii) SBR levels rising faster than 8.5 µmol/L per hour in combination with one SBR measurement above the ET threshold or with clinical symptoms of ABE; iii) a clinical diagnosis of ABE.[(15)]

Confidential: For Review Only

In the absence of scoring system for neurological symptoms, a simplified ABE definition was used and consisted of two or more symptoms mentioned in the chart ('quiet', 'sleepy', 'less sucking', 'irritable', 'lethargic', 'floppy', 'hypotonic', 'rigid', 'apnoea', 'seizure', 'kernicterus') occurring within two days of the SBR peak.[21]

A computerised formula based on the NICE threshold graphs was used to select charts with SBR measurements fitting inclusion criteria. Clinical charts and originally drawn SBR trajectories were cross-checked to confirm eligibility. The first author and a paediatrician (LT) reviewed clinical charts for classifying available neurological symptoms, collecting ET referral and possible cause of death information.

Three available factors aggravating the risk of ABE other than prematurity (ABO incompatibility; G6PD deficiency measured by fluorescent spot test;[22] neonatal sepsis) were extracted from computerised databases. Clinical diagnoses followed the local consensus-based SMRU neonatal guidelines. EGA <35 weeks defined prematurity in this cohort as it is commonly used to assess the risk of extreme NH in the literature.[3, 12, 23, 24] In this setting most pregnancies have EGA calculated by ultrasound[25] or by the best estimate of gestation available if late presentation to antenatal care.[26]

Neurodevelopmental assessment

Children with extreme NH discharged alive from SCBU were traced using their parents address. For those consenting to participate, a one-off appointment was organised at the nearest SMRU clinic between November 2016 and March 2017. Medical history extracted from the child's medical booklet, clinical, and neurological examination findings were reported on a standardised form. Maternal concern about the child's well-being was evaluated by asking whether the child had difficulty seeing, hearing, speaking, bathing, dressing, or in terms of mobility, and reported as yes/no. The Griffiths Mental Development

Confidential: For Review Only

Scales-Extended revised (GMDS-ER) was used to assess neuro-cognitive development.[27] Test items were scored as 'pass' or 'fail' on six individual subscales and percentile scores were derived from raw scores. A general neurodevelopment score EGA-adjusted was calculated per GMDS-ER Administration and Analysis Manuals and categorized as; poor (<10th percentile), delayed (10-49th percentiles) and normal (50th percentile or above). Visual function was assessed with the Cardiff cards for visual acuity and visual contrast.[28] All tests were performed by local staff trained and regularly quality-controlled in clinical neurological examination and neurodevelopmental testing.

Comparator group

A key part of this project involved methodology to permit clinical and neurodevelopmental outcomes with a valid comparator group. Children from the same population born at SMRU in the same year; who had no neurological signs in the neonatal period; and any SBR measurements at least 50 µmol/L below the ET threshold, were identified by retrospective review of clinical charts. As a high proportion of untraceable potential participants was expected, invitations to parents in the comparator group were only made when an extreme NH child was booked for their appointment. The first comparator children to present were included in the analysis but if two made the effort to come to the clinic on the same day, both were accepted and tested.

Statistics

Analysis was performed using IBM Corp. Released 2016. IBM SPSS Statistics for Macintosh, Version 24.0.0.1 Armonk, NY: IBM Corp. Continuous data were described by their median and Inter Quartile Range (IQR) and compared using Mann-Whitney test. Categorical data were described by their proportion and compared with Chi-square test, Fisher's exact test or

Confidential: For Review Only

Chi-square test for trend as appropriate. Factors associated with ABE were evaluated in univariate analysis and reported as Odds-Ratio (OR) with 95% Confidence Interval (CI).

Ethics approval

The study was approved by the Oxford Tropical Research Ethics Committee (OXTREC 5113-16) and the Ethics Committee of the Faculty of Tropical Medicine of the Mahidol University (TMEC 16-071). The Tak Province Border Community Ethics Advisory Board representing the local community reviewed and accepted the study protocol (TCAB-01/REV/2016).

Results

Between 2009 and 2014 SMRU birthing units delivered 12,948 livebirths ≥28 weeks EGA; 2,980 (23.0%) were hospitalised in the SCBU; among them 1,946 were diagnosed with jaundice and 129 fulfilled the criteria of extreme NH, **Figure 2**. This represented an estimated extreme NH incidence of 99.6 per 10,000 livebirths (95%CI: 83.2–118.3).

General characteristics of these 129 neonates are summarised in **Table 1**. The majority (70.5%) were from the refugee camp as expected given the longer data period, almost half (46.5%) were born from primigravid mothers. Sepsis was clinically diagnosed prior to onset of NH in 26.4% of hospitalisations.

The median (IQR) value of SBR peak was 430 (371–487) µmol/L and occurred at a median 97 (68–136) hours of life. Five neonates were referred; four successfully underwent ET, one died of sepsis without confirmation of ET being attempted.

Mortality and ABE

Extreme NH case fatality rate was 10.9% giving a mortality rate of 108.1 per 100,000 livebirths (95%CI: 59.1–181.4); neurological symptoms compatible with ABE were present in

Confidential: For Review Only

28.2% of the clinical charts of neonates not transferred for ET (35/124). All deaths occurred among neonates with ABE.

The odds of developing ABE was higher among neonates born <35 weeks EGA (41.6%, 15/36, vs 22.73%, 20/88, OR 2.4 (95%CI: 1.1-5.7)) or treated for clinical sepsis prior to onset of NH (51.6%, 16/31, vs 20.4%, 19/93, OR 4.2 (95%CI 1.8-9.9)) but not for those with ABO incompatibility or G6PD deficiency.

Long-term outcomes

Thirty-seven children survivors of extreme NH were present for neurodevelopmental assessment, including three who received ET. Neonatal characteristics of the 37 evaluated and the 78 untraceable children were similar (data not shown). The comparator group of 39 children was of similar gender, age and family status however there were fewer neonates born <35 weeks, length of SCBU hospitalisation was shorter and their mothers were more often literate (**Table 2**).

Clinical, visual, neurological and neurodevelopmental outcomes are presented in **Table 3**. While none of the children seen were acutely ill, 25.0% reported more than one hospitalization per year. The proportion of chronic malnutrition was high (30.3% of children stunted) and half of the participants had teeth damage with black staining or cavities. Abnormal visual examination was reported in 18.4% children mostly related to poor acuity. None of the children wore glasses.

Two survivors of extreme NH (5.4%) had an abnormal neurological exam, one compatible with kernicterus: up- and downward gaze paralysis, dystonia and suspected left-sided hearing loss, the other with cerebral palsy. Both had general neurodevelopment scores below the 1st percentile.

Confidential: For Review Only

Median (IQR) percentile scores in all GMDS subscales were poorer among the survivors of extreme NH (11 (2-44) vs 31 (4-67)); difference was more pronounced in the subscale ‘Practical Reasoning’ (**Table 3**). However four survivors of extreme NH (10.8%) had a general percentile score above the 50th centile, the highest score reaching the 82th centile.

Discussion

This study reports the immediate outcomes and long-term consequences of extreme NH in a limited-resource setting. Reflecting on the burden estimates for South-East Asia of 251.3 per 10,000 livebirths and the worldwide 1,309 deaths per 100,000 livebirths presented in the publications by Slusher[8] and Olusanya[9] both the incidence of extreme NH and its mortality rate during this 6-year evaluation are reassuringly low. This likely reflects the benefit of onsite SBR measurements and thus an early phototherapy initiation. The case fatality rate among neonates with extreme NH treated with phototherapy was similar to that described in tertiary paediatric referral hospitals in Myanmar.[29]

Clinical symptoms compatible with ABE were present in 28.2% of neonates, a proportion within the range reported in the literature[3, 30] but with deleterious consequences: all deaths occurred among neonates with ABE and of the two children with severe neurological impairment at least one was suspect to be ABE sequelae. Routine recording of clinical symptoms using a system such as the Bilirubin-Induced Neurological Dysfunction scales might help capture earlier neurological abnormalities and enhance closer monitoring alongside the use of dynamic treatment thresholds.[21] This, however, could only be of added benefit if timely referral can be organized as well. As a first step, emphasizing the need of systematic SBR monitoring, ensuring regular feeds during phototherapy, and verifying that

Confidential: For Review Only

adequate irradiance levels are provided are achievable recommendations to minimize ABE risk.[18, 31]

The one-off neurodevelopmental evaluation's results of survivors of extreme NH ranged from severely impaired to developmentally normal consistent with current published rates.[7] Overall their test performance was poorer than that of a group of children comparable in age, gender and family status. This was particularly observed during practical reasoning, an indispensable skill if one wants to perform adequately at school, and to a lesser extend in language development.

The clinical findings of this study confirm the harsh environment in which those children thrive: a high burden of chronic malnutrition, infections, poor visual and dental care.[32-36] Moderate to severe delay in development after surviving extreme NH will add to an already heavy burden and to the worries of families. In such a precarious socio-economic environment, caregivers of neonates with extreme NH should be encouraged to attend a baby clinic to actively identify and treat nutritional and clinical problems influencing growth and development.

This study's results might not be extrapolated elsewhere due to its limitations. ABE symptoms thought to be representative of abnormal neurological condition were extracted from the daily medical notes and the lack of a systematic checklist means that the proportion of ABE might have been underestimated. The absence of regular follow-up after discharge from the SCBU limited the search for survivors 2-8 years later to those families who remained in the area and possibly under-estimated the mortality rate while overestimating the proportion of children with severe delayed development if one assumes the family would remain closest to what little health care services are available. However the similar proportion of chronic malnutrition, reports of being unwell, poor oral hygiene and

Confidential: For Review Only

visual acuity between survivors of extreme NH and children otherwise comparable in age, gender and family status, suggest that the delayed development observed might be real despite a too small sample size to allow a meaningful statistical analysis. In addition, differences were more pronounced in domains related to language and reasoning skills, both plausibly affected by bilirubin neurotoxicity.[7, 37] Neurodevelopmental tests were performed by locally trained staff fluent in local languages rather than by specialists and cultural and environmental factors could have influenced the overall poor test outcomes. Nevertheless all children came from the same environment, school exposure was generally limited, the health staff was routinely quality controlled to report the pass/fail items as per test guideline and was unaware of the final scoring.

Conclusion

Extreme NH is a serious neonatal health issue in this low-resource setting and possibly contributes to delayed development of children already vulnerable to a heavy burden of chronic malnutrition and infections. Early identification and adequate treatment of NH where ET is not readily available is key to minimize the risk of extreme SBR values or neurological symptoms; additional efforts are necessary to encourage parents to attend the baby clinic to identify and treat early concomitant health and nutritional problems.

Acknowledgements

We thank all the SMRU staff, and the parents and children who were willing to join in the study. Thanks to Stepping Stones physiotherapy for caring for the disabled children.

Confidential: For Review Only

What is already known on this topic:

- Extreme values of serum bilirubin (SBR) in the neonatal period can lead to death and long-term disability.

- Subtle disabilities and neurodevelopment delay might occur after recovery of neonatal hyperbilirubinemia.

- Low- and middle-income countries bear the highest burden of disease, but have limited access to diagnosis, treatment and supportive care for disability.

What this study adds:

- A large majority of neonates with extreme SBR levels survive after onsite phototherapy but limited access to exchange transfusion.

- Long term neurodevelopmental impairments, more pronounced in 'reasoning skills' and 'language', affect survivors already bearing a heavy burden of chronic malnutrition and infections.

- Early recognition and adequate treatment is key to minimize risk of extreme SBR values or neurological symptoms.

Confidential: For Review Only

References

1. Bhutani VK, Johnson-Hamerman L. The clinical syndrome of bilirubin-induced neurologic dysfunction. *Semin Fetal Neonatal Med*. 2015;20(1):6-13.
2. Bhutani VK, Wong RJ, Stevenson DK. Hyperbilirubinemia in Preterm Neonates. *Clin Perinatol*. 2016;43(2):215-32.
3. Gamaleldin R, Iskander I, Seoud I, et al. Risk factors for neurotoxicity in newborns with severe neonatal hyperbilirubinemia. *Pediatri*cs. 2011;128(4):e925-31.
4. Watchko JF, Tiribelli C. Bilirubin-induced neurologic damage--mechanisms and management approaches. *N Engl J Med*. 2013;369(21):2021-30.
5. Weng YH, Chiu YW, Cheng SW, et al. Risk assessment for adverse outcome in term and late preterm neonates with bilirubin values of 20 mg/dL or more. *Am J Perinatol.* 2011;28(5):405-12.
6. Shapiro SM. Chronic bilirubin encephalopathy: diagnosis and outcome. *Semin Fetal Neonatal Med*. 2010;15(3):157-63.
7. Wusthoff CJ, Loe IM. Impact of bilirubin-induced neurologic dysfunction on neurodevelopmental outcomes. *Semin Fetal Neonatal Med*. 2015;20(1):52-7.
8. Slusher TM, Zamora TG, Appiah D, et al. Burden of severe neonatal jaundice: a systematic review and meta-analysis. *BMJ Paediatr Open*. 2017;1(1):e000105.
9. Olusanya BO, Teeple S, Kassebaum NJ. The Contribution of Neonatal Jaundice to Global Child Mortality: Findings From the GBD 2016 Study. *Pediatrics*. 2018;141(2).
10. Bhutani VK, Johnson L. Kernicterus in late preterm infants cared for as term healthy infants. *Semin Perinatol*. 2006;30(2):89-97.

Confidential: For Review Only

11. Bhutani VK, Zipursky A, Blencowe H, et al. Neonatal hyperbilirubinemia and Rhesus disease of the newborn: incidence and impairment estimates for 2010 at regional and global levels. *Pediatr Res*. 2013;74 Suppl 1:86-100.

12. Mukhopadhyay K, Chowdhary G, Singh P, et al. Neurodevelopmental outcome of acute bilirubin encephalopathy. *J Trop Pediatr*. 2010;56(5):333-6.

13. Sgro M, Campbell DM, Kandasamy S, et al. Incidence of chronic bilirubin encephalopathy in Canada, 2007-2008. *Pediatrics*. 2012;130(4):e886-90.

14. American Academy of Pediatrics Subcommittee on H. Management of hyperbilirubinemia in the newborn infant 35 or more weeks of gestation. *Pediatrics*. 2004;114(1):297-316.

15. NICE. Neonatal jaundice: clinical guideline. London: National Collaborating Centre for Women's and Children's Health; 2010. https://www.nice.org.uk/guidance/cg98/evidence/full-guideline-245411821 (Accessed 30 December 2019).

16. Olusanya BO, Emokpae AA. Use of Transcutaneous Bilirubin to Determine the Need for Phototherapy in Resource-Limited Settings. *Neonatology*. 2017;111(4):324-30.

17. Turner C, Carrara V, Aye Mya Thein N, et al. Neonatal intensive care in a Karen refugee camp: a 4 year descriptive study. *PLoS One*. 2013;8(8):e72721.

18. Thielemans L, Trip-Hoving M, Landier J, et al. Indirect neonatal hyperbilirubinemia in hospitalized neonates on the Thai-Myanmar border: a review of neonatal medical records from 2009 to 2014. *BMC Pediatr*. 2018;18(1):190.

19. Mabogunje CA, Olaifa SM, Olusanya BO. Facility-based constraints to exchange transfusions for neonatal hyperbilirubinemia in resource-limited settings. *World J Clin Pediatr*. 2016;5(2):182-90.

Confidential: For Review Only

20. Sanpavat S. Exchange transfusion and its morbidity in ten-year period at King Chulalongkorn Hospital. *J Med Assoc Thai*. 2005;88(5):588-92.

21. Radmacher PG, Groves FD, Owa JA, et al. A modified Bilirubin-induced neurologic dysfunction (BIND-M) algorithm is useful in evaluating severity of jaundice in a resource-limited setting. *BMC Pediatr*. 2015;15:28.

22. Thielemans L, Gornsawun G, Hanboonkunupakarn B, et al. Diagnostic performances of the fluorescent spot test for G6PD deficiency in newborns along the Thailand-Myanmar border: A cohort study. *Wellcome Open Res.* 2018;3:1.

23. Ebbesen F, Andersson C, Verder H, et al. Extreme hyperbilirubinaemia in term and near-term infants in Denmark. *Acta Paediatr*. 2005;94(1):59-64.

24. Kuzniewicz MW, Wickremasinghe AC, Wu YW, et al. Incidence, etiology, and outcomes of hazardous hyperbilirubinemia in newborns. *Pediatrics*. 2014;134(3):504-9.

25. Rijken MJ, Mulder EJ, Papageorghiou AT, et al. Quality of ultrasound biometry obtained by local health workers in a refugee camp on the Thai-Burmese border. *Ultrasound Obstet Gynecol*. 2012;40(2):151-7.

26. Moore KA, Simpson JA, Thomas KH, et al. Estimating Gestational Age in Late Presenters to Antenatal Care in a Resource-Limited Setting on the Thai-Myanmar Border. *PLoS One*. 2015;10(6):e0131025.

27. Barnett AL, Guzzetta A, Mercuri E, et al. Can the Griffiths scales predict neuromotor and perceptual-motor impairment in term infants with neonatal encephalopathy? *Arch Dis Child*. 2004;89(7):637-43.

28. Adoh TO, Woodhouse JM, Oduwaiye KA. The Cardiff Test: a new visual acuity test for toddlers and children with intellectual impairment. A preliminary report. *Optom Vis Sci*. 1992;69(6):427-32.

Confidential: For Review Only

29. Arnolda G, Nwe HM, Trevisanuto D, et al. Risk factors for acute bilirubin encephalopathy on admission to two Myanmar national paediatric hospitals. *Matern Health Neonatol Perinatol*. 2015;1:22.

30. Salas AA, Mazzi E. Exchange transfusion in infants with extreme hyperbilirubinemia: an experience from a developing country. *Acta Paediatr*. 2008;97(6):754-8.

31. Olusanya BO, Ogunlesi TA, Kumar P, et al. Management of late-preterm and term infants with hyperbilirubinaemia in resource-constrained settings. *BMC Pediatr*. 2015;15:39.

32. Boel M, Carrara VI, Rijken M, P, et al. Complex Interactions between soil-transmitted helminths and malaria in pregnant women on the Thai-Burmese border. *PLoS Negl Trop Dis.* 2010;4(11):e887.

33. Kemmer TM, Bovill ME, Kongsomboon W, et al. Iron deficiency is unacceptably high in refugee children from Burma. *J Nutr*. 2003;133(12):4143-9.

34. Swe ZY, Narksawat K, Chansatitporn N. Dental caries status and oral health behavior among Myanmar migrant workers in Mae Sot district, Tak Province, Thailand. Myanmar *Dental Journal*. 2016. http://www.mda-myanmar.org/wp-content/uploads/2015/01/Dental-Caries-Status-and-Oral-health-behaviour.pdf (Accessed 30 December 2019).

35. Turner C, Turner P, Carrara V, et al. High rates of pneumonia in children under two years of age in a South East Asian refugee population. *PLoS One*. 2013;8(1):e54026.

36. UNICEF. Situation analysis of children with disabilities in the Republic of the Union of Myanmar. Yangon: UNICEF, Myanmar; 2016. https://reliefweb.int/sites/reliefweb.int/files/resources/Full_report_in_English_0.pdf. (Accessed 30 December 2019).

37. Shapiro SM, Popelka GR. Auditory impairment in infants at risk for bilirubin-induced neurologic dysfunction. *Semin Perinatol*. 2011;35(3):162-70.

Confidential: For Review Only

Table 1: Characteristics of neonatal period of 129 neonates with extreme NH

**Characteristics**		**Extreme NH (n=129)**
Year of admission	2009	22 (17.0%)
	2010	26 (20.2%)
	2011	12 (9.3%)
	2012	24 (18.6%)
	2013	27 (20.9%)
	2014	18 (14.0%)
Status	Refugee	91 (70.5%)
	Migrant	38 (29.5%)
Gender (male)		75 (58.1%)
Gestational age[a] ≥ 35 weeks		92 (71.3%)
Place of delivery	SMRU clinic	110 (85.3%)
	Tertiary hospital	4 (3.1%)
	Home	15 (11.6%)
Birthweight[b] (kg) (n=125) (median, IQR)		2.53 (1.96 – 2.90)
Small for Gestational Age[c] (n=125)		23 (18.4%)
Poor start to life[d]		15 (11.6%)
Mother primigravid		60 (46.5%)
Normal vaginal delivery		114 (88.4%)
Age at admission (days) (median, IQR)		1 (0 – 4)
Duration of admission (days) (median, IQR)		6 (4 – 11)
Duration of phototherapy (hours) (median, IQR)		76 (48 – 124)
Peak SBR (µmol/L) (median, IQR)		430 (371 – 487)
Age at peak SBR (hours) (median, IQR)		97 (68 – 136)
Peak SBR at <72 hours of life		41 (31.8%)
Received exchange transfusion		4 (3.1%)
Potential ABO incompatibility[e] (n=126)		24 (19.0%)
G6PD-deficient male[f] (n=75)		33 (44.0%)
Polycythaemia[g]		10 (7.8%)
Birth trauma (visible bruising after birth recorded in the chart)		6 (4.7%)
Clinical diagnosis of sepsis[h] prior to onset of NH		34 (26.4%)

Data shown in number and (percentage) unless stated otherwise.
[a]Gestational age: Based on first trimester ultrasound.[25]
[b]Birthweight: weight measurement considered valid if done in the first 72 hours of life.
[c]Small for Gestational Age: Defined as birth weight below the 10th percentile for gestational age and sex, calculated with the Intergrowth-21st newborn size application tool (https://intergrowth21.tghn.org).
[d]Poor start to life: Apgar score <7 at 5 minutes and/or clinical suspicion of meconium aspiration and/or resuscitation at birth with at least five inflation breaths.
[e]Potential ABO incompatibility: Mother-fetus pairs with mother blood group "O" and neonate blood group "A" or "B". Rhesus factor and Coombs test were not available.
[f]Diagnosed by G6PD fluorescent test.[22]

Confidential: For Review Only

[g]Polycythaemia: Two consecutive haematocrit values >70% from a capillary sample, or a diagnosis of polycythaemia recorded in the clinical chart.

[h]Clinical diagnosis of sepsis: sepsis reported as clinical diagnosis treated at least 5 days by intravenous antibiotics (blood culture confirmations were not available).

Confidential: For Review Only

388 **Table 2: Neonatal period characteristics of 37 survivors of extreme NH and a comparator**
389 **group of 39 children**

Characteristics	Extreme NH (n=37)	Comparator Group (n=39)	p-value
Gender (male)	20 (54.1%)	21 (53.8%)	0.985
Age (months) (median, IQR)	51 (35 – 81)	52 (36 – 83)	0.938
Refugee status	23 (62.1%)	25 (64.1%)	0.861
First born	20 (54.1%)	20 (51.3%)	0.990
EGA (weeks + days) (median, IQR)	37+4 (34+5 – 38+3)	39+5 (37+5 – 40+1)	<0.001
EGA <35 weeks	11 (29.7%)	4 (10.3%)	0.033
Small for gestational age	5 (13.5%)	3 (7.7%)	0.383
Normal vaginal delivery	31 (83.8%)	36 (92.3%)	0.097
Days in SCBU (median, IQR)	8 (5 – 16)	4 (2 – 5)	<0.001
Maternal age (median, IQR)	24 (20 – 30)	24 (19 – 30)	0.988
Maternal illiteracy	9/27 (33.3%)	4/34 (11.8%)	0.041
Maternal smoking	7 (18.9%)	4 (10.3%)	0.283

390 Data shown in number and (percentage) unless stated otherwise.

Confidential: For Review Only

Table 3. Long term clinical, visual, neurological, and neurodevelopmental outcomes of 37 survivors of extreme NH and of a comparator group of 39 children

	Extreme NH (n=37)	Comparator Group (n=39)	p-value
**Clinical outcome**			
Unwell-being[a]	11/36 (30.6%)	8 (20.5%)	0.318
Stunting[b]	15 (40.5%)	8 (20.5%)	0.057
Teeth abnormality[c]	18/36 (50.0%)	21/38 (55.3%)	0.650
**Visual and neurological outcomes**			
Visual examination abnormality[d]	8 (21.6%)	6 (15.4%)	0.483
Neurological exam abnormality[e]	2 (5.4%)	0	0.141
**Neurodevelopmental outcome**			
Maternal perception of child's development: mother has worries	6 (16.2%)	2 (5.1%)	0.115
**GMDS scores**			
General percentile score (median, IQR)	11 (2 – 42)	31 (4 – 67)	0.025
Poor development (<10th centile)	16 (43.2%)	11 (28.2%)	
Delayed development (10-49th centile)	17 (46.0%)	13 (33.3%)	
Normal development (≥50th centile)	4 (10.8%)	15 (38.5%)	0.020
Locomotion (median, IQR)	19 (5 – 58)	46 (13 – 65)	0.173
Personal and social (median, IQR)	26 (11 – 64)	46 (20 – 72)	0.333
Hearing and language (median, IQR)	11 (<1 – 33)	27 (4 – 62)	0.078
Eye and hand co-ordination (median, IQR)	22 (4 – 46)	29 (5 – 44)	0.460
Performance (median, IQR)	4 (<1 – 40)	15 (3 – 69)	0.147
Practical reasoning (median, IQR)[f]	14 (2 – 49)	50 (10 – 67)	0.013

Data shown in number, and (percentage).

[a]Unwell-being: more than one hospitalization per year or presence of a chronic disease as reported in the child's medical booklet.

[b]Stunting: height-for-age z-scores below -2 as calculated with WHO Child Growth Standards (http://www.who.int/childgrowth/en/).

[c]Teeth abnormality: judgement of medical staff looking for the presence of black staining or cavities, confirmed by first author through photographic records.

[d]Visual examination abnormality: unconjugated eye movement and Cardiff's binocular visual contrast and visual acuity lower than the normal values by age categories (instructions manual for the Cardiff Acuity Test).

[e]Neurological examination abnormality: judgement of the medical staff after general observation while moving, and evaluating tone, strength and sensibility, coordination, gait and posture, and reflexes.

[f]Two children had an adjusted age < 2years old when tested, therefore practical reasoning was only available for 36 children with extreme NH and 38 in the comparator group.

Confidential: For Review Only

Figures

Figure 1: Location of the three Shoklo Malaria Research Unit (SMRU) clinics.

The three clinics are: Mae La clinic for refugees; Wang Pha and Maw Ker Thai clinics for migrant population. SMRU main office is located in Mae Sot town.

Photo credit: Daniel Parker.

Figure 2: Flowchart representing the selection of neonates with extreme hyperbilirubinemia (NH).

[a]Major congenital abnormality was defined as an abnormal finding on surface examination and/or auscultation of the heart and lungs reported in the medical chart and confirmed by a medical doctor.

Confidential: For Review Only

Competing interests

The authors have declared that no competing interests exist.

Patient and Public Involvement

The Tak Province Border Community Ethics Advisory Board members representing the local community where the study took place reviewed the protocol and their input into the design of the project was taken into account.

Funding

Shoklo Malaria Research Unit is part of the Mahidol-Oxford University Tropical Medicine Research Program at the Faculty of Tropical Medicine, Mahidol University, Bangkok, Thailand, and supported by the Wellcome Trust of Great Britain. The funders had no role in study design, data collection and analysis, decision to publish, or preparation of the manuscript. LT was supported by a PhD grant from 'The Belgian Kids' Fund for Pediatric Research.

Contribution

EMNW, LT, VIC conceived and designed the study, and BH, PFR, CT, FHN and RM contributed to the study design. EMNW, LT and VIC coordinated the study and supervised the research teams. MCD, AAN, WS, SK performed all the neurodevelopment tests and clinical examinations. TN developed the data collection and quality controlled. EMNW, LT and VIC led the analysis and interpretation of the data with JL leading statistical analysis. All authors supported the preparation and later critically reviewed the manuscript including in the final approval of the manuscript. All authors had full access to all of the data (including statistical

Confidential: For Review Only

reports and tables) in the study and can take responsibility for the integrity of the data and the accuracy of the data analysis.

Confidential: For Review Only

Confidential: For Review Only

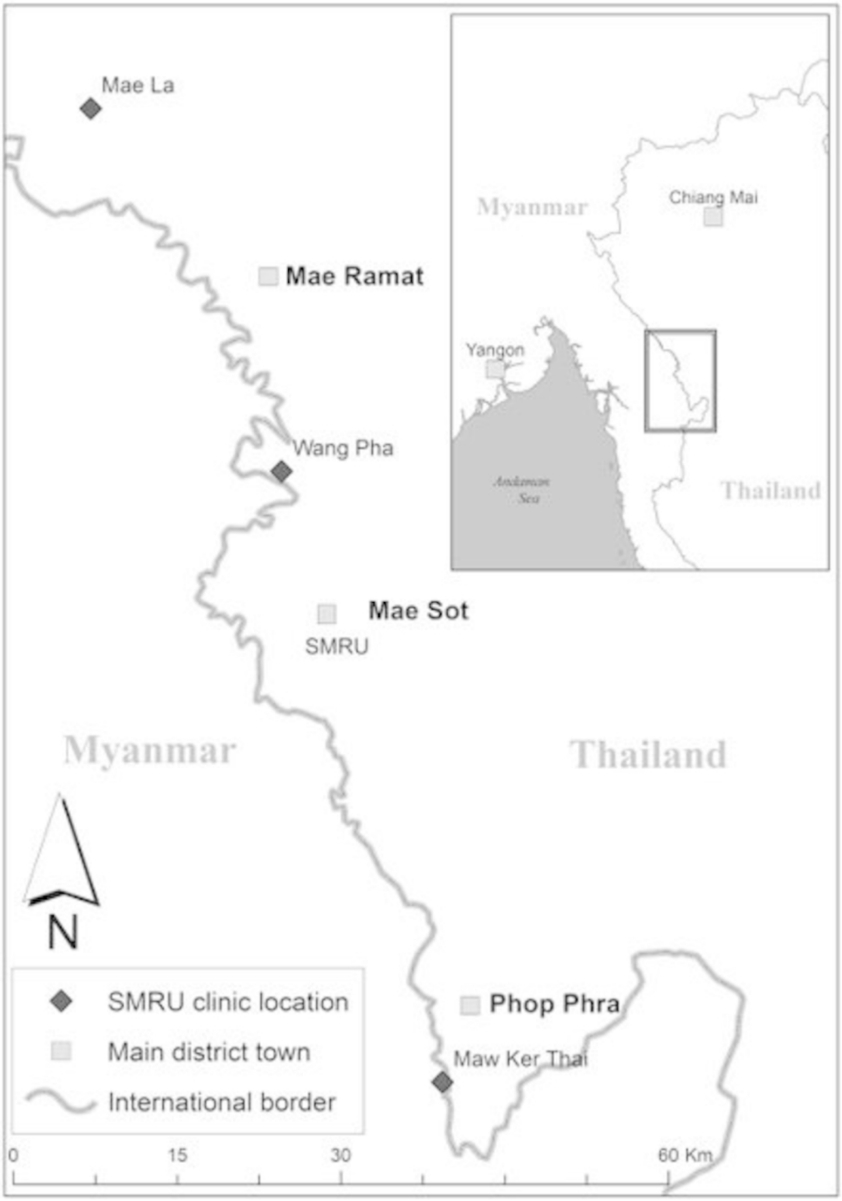

115x163mm (300 x 300 DPI)

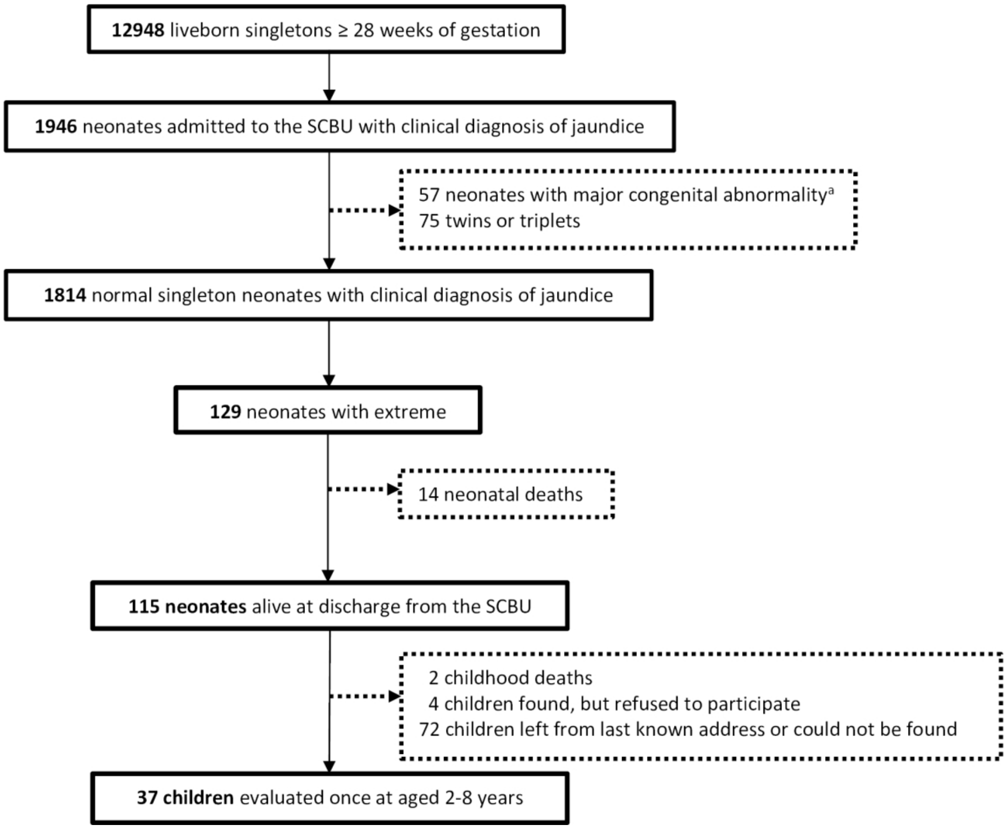

166x136mm (300 x 300 DPI)

BMJ Paediatrics Open

EXTREME NEONATAL HYPERBILIRUBINEMIA IN REFUGEE AND MIGRANT POPULATIONS: RETROSPECTIVE COHORT

Journal:	*BMJ Paediatrics Open*
Manuscript ID	bmjpo-2020-000641.R1
Article Type:	Original research
Date Submitted by the Author:	14-Apr-2020
Complete List of Authors:	Wouda, Eva; Shoklo Malaria Research Unit; University of Groningen, University Medical Center<br>Thielemans, Laurence; Shoklo Malaria Research Unit; Universite Libre de Bruxelles, Neonatology-Pediatrics, Hôpital Erasme<br>Darakamon, Mue; Shoklo Malaria Research Unit<br>Nge, Aye; Shoklo Malaria Research Unit<br>Say, Wah; Shoklo Malaria Research Unit<br>Khing, Sanda; Shoklo Malaria Research Unit<br>Hanboonkunupakarn, Borimas; Mahidol Oxford Tropical Medicine Research Unit, Faculty of Tropical Medicine<br>Ngerseng, Thatsanun ; Mahidol Oxford Tropical Medicine Research Unit, Faculty of Tropical Medicine<br>Landier, Jordi; Shoklo Malaria Research Unit; Universite Aix Marseille, IRD, INSERM, SESSTIM<br>van Rheenen, Patrick; University Medical Center Groningen, Paediatric Gastroenterology<br>Turner, Claudia; Cambodia Oxford Medical Research Unit; University of Oxford Centre for Tropical Medicine and Global Health, Nuffield Department of Medicine<br>Nosten, Francois; Shoklo Malaria Research Unit; University of Oxford Centre for Tropical Medicine and Global Health, Nuffield Department of Medicine<br>McGready, Rose; Shoklo Malaria Research Unit; University of Oxford Centre for Tropical Medicine and Global Health, Nuffield Department of Medicine<br>Carrara, Verena; Shoklo Malaria Research Unit; Swiss Tropical and Public Health Institute, Department of Medicine
Keywords:	Jaundice, Neurodevelopment, Neurodisability

SCHOLARONE™
Manuscripts

I, the Submitting Author has the right to grant and does grant on behalf of all authors of the Work (as defined in the below author licence), an exclusive licence and/or a non-exclusive licence for contributions from authors who are: i) UK Crown employees; ii) where BMJ has agreed a CC-BY licence shall apply, and/or iii) in accordance with the terms applicable for US Federal Government officers or employees acting as part of their official duties; on a worldwide, perpetual, irrevocable, royalty-free basis to BMJ Publishing Group Ltd ("BMJ") its licensees and where the relevant Journal is co-owned by BMJ to the co-owners of the Journal, to publish the Work in this journal and any other BMJ products and to exploit all rights, as set out in our licence.

The Submitting Author accepts and understands that any supply made under these terms is made by BMJ to the Submitting Author unless you are acting as an employee on behalf of your employer or a postgraduate student of an affiliated institution which is paying any applicable article publishing charge ("APC") for Open Access articles. Where the Submitting Author wishes to make the Work available on an Open Access basis (and intends to pay the relevant APC), the terms of reuse of such Open Access shall be governed by a Creative Commons licence – details of these licences and which Creative Commons licence will apply to this Work are set out in our licence referred to above.

Other than as permitted in any relevant BMJ Author's Self Archiving Policies, I confirm this Work has not been accepted for publication elsewhere, is not being considered for publication elsewhere and does not duplicate material already published. I confirm all authors consent to publication of this Work and authorise the granting of this licence.

Confidential: For Review Only

EXTREME NEONATAL HYPERBILIRUBINEMIA IN REFUGEE AND MIGRANT POPULATIONS: RETROSPECTIVE COHORT

Eva M N Wouda[1,2], Laurence Thielemans[1,3], Mue C Darakamon[1], Aye A Nge[1], Wah Say[1], Sanda Khing[1], Borimas Hanboonkunupakarn[4], Thatsanun Ngerseng[4], Jordi Landier[1,5], Patrick F van Rheenen[2], Claudia Turner[6,7,8], Francois H Nosten[1,4,6], Rose McGready[1,4,6], Verena I Carrara[1,9]

[1]Shoklo Malaria Research Unit, Faculty of Tropical Medicine, Mahidol University, Mae Sot, Thailand;

[2]University of Groningen, University Medical Center Groningen, the Netherlands;

[3]Neonatology-Pediatrics, Cliniques Universitaires de Bruxelles - Hôpital Erasme, Université Libre de Bruxelles, Belgium;

[4]Mahidol-Oxford Tropical Medicine Research Unit (MORU), Faculty of Tropical Medicine, Mahidol University;

[5]Aix Marseille University, IRD, INSERM, SESSTIM, Marseille, France;

[6]Centre for Tropical Medicine and Global Health, Nuffield Department of Medicine, University of Oxford;

[7]Cambodia-Oxford Medical Research Unit, Angkor Hospital for Child, Siem Reap, Cambodia;

[8]Angkor Hospital for Children, Siem Reap, Cambodia;

[9]Department of Medicine, Swiss Tropical and Public Health Institute, Basel, Switzerland

Corresponding author: Verena I. Carrara, verena@shoklo-unit.com, +66 (0)55532026

Confidential: For Review Only

Keywords: neonatal hyperbilirubinemia, neonatal jaundice, extreme neonatal hyperbilirubinemia, kernicterus, neurological outcome, neurodevelopment, limited-resource setting, low- and middle-income countries, refugee, migrant

Confidential: For Review Only

Abstract

Objectives To describe neonatal survival and long-term neurological outcome in neonatal hyperbilirubinemia (NH) with extreme serum bilirubin (SBR) values.

Design Retrospective chart review; a one-off neurodevelopmental evaluation.

Setting Special Care Baby Unit in a refugee camp and clinics for migrant populations at the Thailand-Myanmar border with phototherapy facilities, but limited access to exchange transfusion.

Patients Neonates ≥ 28 weeks of gestational age with extreme SBR values and/or acute neurological symptoms; neurodevelopment evaluation conducted at 23-97 months of age.

Main outcome measures Neonatal mortality rate; prevalence of acute bilirubin encephalopathy (ABE) signs; prevalence of delayed development scores based on the Griffiths Mental Development Scale (GMDS).

Results From 2009 to 2014, 1,946 neonates were diagnosed with jaundice; 129 (6.6%) had extreme SBR values during NH (extreme NH). In this group median peak SBR (inter-quartile-range (IQR)) was 430 (371-487) µmol/L and prevalence of ABE was 28.2%. Extreme NH-related mortality was 10.9% (14/129). Median percentile GMDS-general score of 37 survivors of extreme NH was poor: 11 (2-42). 'Performance', 'Practical reasoning' and 'Hearing & Language' domains were most affected. Four (10.8%) extreme NH survivors had normal development score (≥50th centile). Two (5.4%) developed the most severe form of Kernicterus Spectrum Disorder.

Conclusions In this limited-resource setting poor neonatal survival and neurodevelopmental outcomes, after extreme NH, were high. Early identification and adequate treatment of NH

Confidential: For Review Only

where exchange transfusion is not readily available is key to minimizing the risk of extreme SBR values or neurological symptoms.

Confidential: For Review Only

Background

In the most severe form of neonatal hyperbilirubinemia (NH), serum bilirubin (SBR) levels rise to extreme values causing a life-threatening condition that can lead to lifelong disability.[1] Acute Bilirubin Encephalopathy (ABE) happens during the early phase of the disease producing a vast array of neurological symptoms from mild and reversible to severe and irreversible. Its physiopathology is complex, however prematurity, sepsis, Glucose-6-phosphate dehydrogenase (G6PD) deficiency and ABO/ Rhesus incompatibility contribute to its development.[2-5] Neurological sequelae might progress to kernicterus spectrum disorders (KSD) ranging from subtle to severe motor and/or auditory dysfunction[6] as well as language delay, attention disorders or lower executive function capacities[7].

A review of the worldwide burden of severe NH published in 2017 reported an incidence of 251.3 per 10,000 livebirths in South-East Asia the second highest after Africa.[8] NH-related mortality is high: 1,309 deaths per 100,000 live births in 2016.[9] Low- and middle-income countries bear the heaviest burden; two thirds of deaths and severe sequelae occur in Sub-Saharan Africa and South Asia, 3 to 20% of survivors have normal long-term development.[10-13]

Most NH-related research follows the American Academy of Pediatrics guideline for neonates >35 weeks estimated gestational age (EGA) in which SBR levels >25 mg/dl (428 µmol/L) at age >72 hours are considered extreme and require exchange transfusion (ET).[14] The British National Institute for Health and Clinical Excellence (NICE) guideline proposes dynamic treatment thresholds based on postnatal age and varying with gestational ages from 23 to ≥38 weeks.[15] SBR values considered extreme therefore start as low as 13 mg/dl (230 µmol/L) for the youngest EGA. However publications reporting outcomes of neonates treated following these dynamic treatment thresholds are still rare.[16]

Confidential: For Review Only

The Shoklo Malaria Research Unit (SMRU) set up a Special Care Baby Unit (SCBU) in 2008 in Mae La, the most populous refugee camp along the Thailand-Myanmar border, to provide basic care for neonates including oxygen, intravenous antibiotics, nasogastric feeding, phototherapy and basic laboratory tests.[17] Starting 2011, another two SCBU's were set up in clinics for migrant populations. Care for neonates with NH improved over time following the implementation of standardized guidelines based on the NICE,[15] systematic heel prick SBR measurements with onsite bilirubinometer (Pfaff Medical Bilimeter 2 and 3) and irradiance levels optimization with the introduction of LED phototherapy units.[18] In livebirths from 2009-2014 NH prevalence was 18%; 9% of these neonates had at least one SBR value exceeding the NICE threshold at which ET should be considered.[18] ET is an intervention with potentially serious complications performed in an intensive care unit rather than a primary health facility.[19, 20] Due to the inherent difficulties of sending a refugee or migrant person to a specialised centre, only some neonates could be referred.

The objective of this review was to evaluate the immediate and long term clinical and neurodevelopmental outcomes of children from a low-resource setting with SBR values rising to levels considered extreme.

Methods

Three SMRU SCBU facilities contributed to the data; in refugees (1st January 2009–31st December 2014,[17]), and in migrants (1st January 2011–31st December 2014), **Figure 1.**

Inclusion criteria

Clinical charts of all liveborn singletons ≥28 weeks EGA without major congenital abnormalities born to mothers attending SMRU antenatal care admitted for a clinical diagnosis of jaundice were reviewed.

Definitions

Confidential: For Review Only

For this analysis, NH with SBR values rising above levels that could justify ET, reported in the text as ‘extreme NH’ was defined as either i) two consecutive SBR measurements above the ET threshold of the NICE guideline; ii) SBR levels rising faster than 8.5 µmol/L per hour in combination with one SBR measurement above the ET threshold or with clinical symptoms of ABE; iii) a clinical diagnosis of ABE.[15]

In the absence of scoring system for neurological symptoms, a simplified ABE definition was used and consisted of two or more symptoms mentioned in the chart (‘quiet’, ‘sleepy’, ‘less sucking’, ‘irritable’, ‘lethargic’, ‘floppy’, ‘hypotonic’, ‘rigid’, ‘apnoea’, ‘seizure’, ‘kernicterus’) occurring within two days of the SBR peak.[21]

A computerised formula based on the NICE threshold graphs was used to select charts with SBR measurements fitting inclusion criteria. Clinical charts and originally drawn SBR trajectories were cross-checked to confirm eligibility. The first author and a paediatrician (LT) reviewed clinical charts for classifying available neurological symptoms, collecting ET referral and possible cause of death information.

Three available factors aggravating the risk of ABE other than prematurity (ABO incompatibility; G6PD deficiency measured by fluorescent spot test;[22] neonatal sepsis) were extracted from computerised databases. Clinical diagnoses followed the local consensus-based SMRU neonatal guidelines. EGA <35 weeks defined prematurity in this cohort as it is commonly used to assess the risk of extreme NH in the literature.[3, 12, 23, 24] In this setting most pregnancies have EGA calculated by ultrasound[25] or by the best estimate of gestation available if late presentation to antenatal care.[26] SBR and G6PD results were provided in near real-time by locally trained, routinely quality controlled, laboratory technicians.

Neurodevelopmental assessment

Confidential: For Review Only

124 Children with extreme NH discharged alive from SCBU were traced using their parents address. For those consenting to participate, a one-off appointment was organised at the nearest SMRU clinic between November 2016 and March 2017. Medical history extracted from the child's medical booklet, clinical, and neurological examination findings were reported on a standardised form. Maternal concern about the child's well-being was evaluated by asking whether the child had difficulty seeing, hearing, speaking, bathing, dressing, or in terms of mobility, and reported as yes/no. The Griffiths Mental Development Scales-Extended revised (GMDS-ER[(27)]) was used to assess neuro-cognitive development: it spanned the possible age distribution of this cohort and were familiar to the staff who could perform it in their own language. Test items were scored as 'pass' or 'fail' on six individual subscales and percentile scores were derived from raw scores. A general neurodevelopment score EGA-adjusted was calculated per GMDS-ER Administration and Analysis Manuals and categorized as; poor (<10th percentile), delayed (10-49th percentiles) and normal (50th percentile or above). Visual function was assessed with the Cardiff cards for visual acuity and visual contrast.[(28)] All tests were performed by local staff trained and regularly quality-controlled in clinical neurological examination and neurodevelopmental testing.[(29, 30)]

140 *Statistics*

141 Analysis was performed using IBM Corp. Released 2016. IBM SPSS Statistics for Macintosh, Version 24.0.0.1 Armonk, NY: IBM Corp. Continuous data were described by their median and Inter Quartile Range (IQR) and compared using Mann-Whitney test. Categorical data were described by their proportion and compared with Chi-square test, Fisher's exact test or Chi-square test for trend as appropriate. Factors associated with ABE were evaluated in univariate analysis and reported as Odds-Ratio (OR) with 95% Confidence Interval (CI).

147 *Ethics approval*

Confidential: For Review Only

The study was approved by the Oxford Tropical Research Ethics Committee (OXTREC 5113-16) and the Ethics Committee of the Faculty of Tropical Medicine of the Mahidol University (TMEC 16-071). The Tak Province Border Community Ethics Advisory Board representing the local community reviewed and accepted the study protocol (TCAB-01/REV/2016).

Results

Between 2009 and 2014 SMRU antenatal attendees delivered 12,963 singleton congenitally normal livebirths ≥28 weeks EGA; 2,980 (23.0%) were hospitalised in the SCBU; 1,946 were diagnosed with jaundice and 129 fulfilled the criteria of extreme NH, **Figure 2**. Extreme NH incidence among neonates delivered in SMRU birthing units (110/10,398) was estimated at 105.8 per 10,000 livebirths (95%CI: 87.0–127.4).

General characteristics of the 129 neonates are summarised in **Table 1**. The majority (70.5%) were from the refugee camp as expected given the longer data period, almost half (46.5%) were born from primigravid mothers and 37 (29.5%) were <35 weeks EGA. Sepsis was clinically diagnosed prior to onset of NH in 26.4% of hospitalisations.

The median (IQR) value of SBR peak was 430 (371–487) µmol/L and occurred at a median 97 (68–136) hours of life. Five neonates were referred; four successfully underwent ET, one died of sepsis without confirmation of ET being attempted.

Mortality and ABE

Extreme NH case fatality rate was 10.9% giving a mortality rate of 108.1 per 100,000 livebirths (95%CI: 59.1–181.4); neurological symptoms compatible with ABE were reported in 28.2% of the clinical charts of neonates not transferred for ET (35/124). All deaths occurred among neonates with ABE.

Confidential: For Review Only

The odds of developing ABE was higher among neonates born <35 weeks EGA (41.6%, 15/36, vs 22.73%, 20/88, OR 2.4 (95%CI: 1.1-5.7)) or treated for clinical sepsis prior to onset of NH (51.6%, 16/31, vs 20.4%, 19/93, OR 4.2 (95%CI 1.8-9.9)) but not for those with ABO incompatibility or G6PD deficiency.

Long-term outcomes

Thirty-seven children survivors of extreme NH were present for neurodevelopmental assessment, including three who received ET. Their neonatal and maternal characteristics were similar to those of the 78 untraceable children (**Table 2)**.

Clinical, visual, neurological and neurodevelopmental outcomes are presented in **Table 3**. While none of the children seen were acutely ill, 30.6% reported more than one hospitalization per year. The proportion of chronic malnutrition was high (40.5% of children stunted) and half of the participants had teeth damage with black staining or cavities. Abnormal visual examination was reported in 21.6% children mostly related to poor acuity. None of the children wore glasses. Overall children born <35 weeks EGA were reporting similar if not slightly better clinical condition than children born term (differences non-significant).

Two survivors of extreme NH (5.4%), both born at term, had an abnormal neurological examination compatible with a severe form of KSP: one with up- and downward gaze paralysis, dystonia and suspected left-sided hearing loss, the other with cerebral palsy. Their general neurodevelopment score was below the 1st percentile.

The median (IQR) percentile summary score of all GMDS-ER subscales was low (11 (2-42)) with 16 children (43.2%) scoring below the 10th centile; scores were particularly poor in the subscales 'Hearing and language'; 'Performance' and 'Practical Reasoning' (**Table 3**). Differences observed between gestational age groups didn't reach significance level;

Confidential: For Review Only

however children born <35 weeks EGA tended to obtain similar or higher scores than those born term. Four survivors of extreme NH (10.8%) had a general percentile score above the 50th centile, the highest score reaching the 82th centile.

Discussion

This study reports the immediate outcomes and long-term consequences of extreme NH in a limited-resource setting. Reflecting on the burden estimates for South-East Asia of 251.3 per 10,000 livebirths and the worldwide 1,309 deaths per 100,000 livebirths presented in the publications by Slusher(8) and Olusanya(9) both the incidence of extreme NH and its mortality rate during this 6-year evaluation are reassuringly low. This likely reflects the benefit of onsite SBR measurements and thus an early phototherapy initiation. The case fatality rate among neonates with extreme NH treated with phototherapy was similar to that described in tertiary paediatric referral hospitals in Myanmar.(31)

Clinical symptoms compatible with ABE were present in 28.2% of neonates, a proportion within the range reported in the literature(3, 32) but with deleterious consequences: all deaths occurred among neonates with ABE and of the two children with severe neurological impairment at least one was suspect to be ABE sequelae. Routine recording of clinical symptoms using a system such as the Bilirubin-Induced Neurological Dysfunction scales might help capture earlier neurological abnormalities and enhance closer monitoring alongside the use of dynamic treatment thresholds.(21) This, however, could only be of added benefit if timely referral can be organized as well. As a first step, emphasizing the need for systematic SBR monitoring, ensuring regular feeds during phototherapy, and verifying that adequate irradiance levels are provided are achievable recommendations to minimize ABE risk.(18, 33)

Confidential: For Review Only

The one-off neurodevelopmental evaluation results ranged from severely impaired to developmentally normal consistent with current published rates.[7] Test performance was particularly poor in domains related to language and reasoning skills, both plausibly affected by bilirubin neurotoxicity.[6, 7, 34] Reassuringly, survivors of extreme NH <35 weeks EGA performed similarly or even better than those born term despite a higher risk to develop ABE.

Whether children with severe KSP form retain a normal intelligence is debated.[1, 6, 35] As an anecdotic case, and even though it is was not possible to measure intelligence, the single child with features compatible with classic kernicterus appeared frustrated with not being able to complete the proposed tasks.

The clinical findings of this study confirm the harsh environment in which those children thrive: a high burden of chronic malnutrition, infections, poor visual and dental care.[36-40] Moderate to severe delay in development after surviving extreme NH will add to an already heavy burden and to the worries of families. In such a precarious socio-economic environment, caregivers of neonates with extreme NH should be encouraged to attend a baby clinic to actively identify and treat nutritional and clinical problems influencing growth and development, if this is available.

This study's results might not be extrapolated elsewhere due to its limitations. ABE symptoms thought to represent abnormal neurological conditions were extracted from the daily medical notes and the lack of a systematic checklist means that the proportion of ABE might have been underestimated. The absence of regular follow-up after discharge from the SCBU limited the search for survivors 2-8 years later to those families who remained in the area and possibly under-estimated the mortality rate while overestimating the proportion of children with severe delayed development if one assumes the family would remain closest

Confidential: For Review Only

to what little health care services are available. Neurodevelopmental tests were performed by locally trained staff fluent in local languages rather than by specialists and cultural and environmental factors could have influenced the overall poor test outcomes. Nevertheless all children came from the same environment, school exposure was generally limited, the health staff was routinely quality controlled to report the pass/fail items as per test guideline and was unaware of the final scoring.

Conclusion

Extreme NH is a serious neonatal health issue in this low-resource setting and possibly contributes to delayed development of children already vulnerable to a heavy burden of chronic malnutrition and infections. Early identification and adequate treatment of NH where ET is not readily available is key to minimize the risk of extreme SBR values or neurological symptoms; additional efforts are necessary to encourage parents to attend the baby clinic to identify and treat early concomitant health and nutritional problems.

Acknowledgements

We thank all the SMRU staff, and the parents and children who were willing to join in the study. Thanks to Stepping Stones physiotherapy for caring for the disabled children.

Confidential: For Review Only

What is already known on this topic:

- Extreme values of serum bilirubin (SBR) in the neonatal period can lead to death and long-term disability.

- Subtle disabilities and neurodevelopment delay might occur after recovery of neonatal hyperbilirubinemia.

- Low- and middle-income countries bear the highest burden of disease, but have limited access to diagnosis, treatment and supportive care for disability.

What this study adds:

- A large majority of neonates with extreme SBR levels survive after onsite phototherapy but limited access to exchange transfusion.

- Long term neurodevelopmental impairments, more pronounced in 'performance', 'reasoning skills' and 'language', affect survivors already bearing a heavy burden of chronic malnutrition and infections.

- Early recognition and adequate treatment is key to minimize risk of extreme SBR values or neurological symptoms.

Confidential: For Review Only

References

1. Bhutani VK, Johnson-Hamerman L. The clinical syndrome of bilirubin-induced neurologic dysfunction. *Semin Fetal Neonatal Med*. 2015;20(1):6-13.

2. Bhutani VK, Wong RJ, Stevenson DK. Hyperbilirubinemia in Preterm Neonates. *Clin Perinatol*. 2016;43(2):215-32.

3. Gamaleldin R, Iskander I, Seoud I, et al. Risk factors for neurotoxicity in newborns with severe neonatal hyperbilirubinemia. *Pediatrics*. 2011;128(4):e925-31.

4. Watchko JF, Tiribelli C. Bilirubin-induced neurologic damage--mechanisms and management approaches. *N Engl J Med*. 2013;369(21):2021-30.

5. Weng YH, Chiu YW, Cheng SW, et al. Risk assessment for adverse outcome in term and late preterm neonates with bilirubin values of 20 mg/dL or more. *Am J Perinatol*. 2011;28(5):405-12.

6. Le Pichon JB, Riordan SM, Watchko J, et al. The Neurological Sequelae of Neonatal Hyperbilirubinemia: Definitions, Diagnosis and Treatment of the Kernicterus Spectrum Disorders (KSDs). *Curr Pediatr Rev*. 2017;13(3):199-209.

7. Wusthoff CJ, Loe IM. Impact of bilirubin-induced neurologic dysfunction on neurodevelopmental outcomes. *Semin Fetal Neonatal Med*. 2015;20(1):52-7.

8. Slusher TM, Zamora TG, Appiah D, et al. Burden of severe neonatal jaundice: a systematic review and meta-analysis. *BMJ Paediatr Open*. 2017;1(1):e000105.

9. Olusanya BO, Teeple S, Kassebaum NJ. The Contribution of Neonatal Jaundice to Global Child Mortality: Findings From the GBD 2016 Study. *Pediatrics*. 2018;141(2).

10. Bhutani VK, Johnson L. Kernicterus in late preterm infants cared for as term healthy infants. *Semin Perinatol*. 2006;30(2):89-97.

Confidential: For Review Only

11. Bhutani VK, Zipursky A, Blencowe H, et al. Neonatal hyperbilirubinemia and Rhesus disease of the newborn: incidence and impairment estimates for 2010 at regional and global levels. *Pediatr Res*. 2013;74 Suppl 1:86-100.

12. Mukhopadhyay K, Chowdhary G, Singh P, et al. Neurodevelopmental outcome of acute bilirubin encephalopathy. *J Trop Pediatr*. 2010;56(5):333-6.

13. Sgro M, Campbell DM, Kandasamy S, et al. Incidence of chronic bilirubin encephalopathy in Canada, 2007-2008. *Pediatrics*. 2012;130(4):e886-90.

14. American Academy of Pediatrics Subcommittee on H. Management of hyperbilirubinemia in the newborn infant 35 or more weeks of gestation. *Pediatrics*. 2004;114(1):297-316.

15. NICE. Neonatal jaundice: clinical guideline. London: National Collaborating Centre for Women's and Children's Health; 2010. https://www.nice.org.uk/guidance/cg98/evidence/full-guideline-245411821 (Accessed 30 December 2019).

16. Olusanya BO, Emokpae AA. Use of Transcutaneous Bilirubin to Determine the Need for Phototherapy in Resource-Limited Settings. *Neonatology*. 2017;111(4):324-30.

17. Turner C, Carrara V, Aye Mya Thein N, et al. Neonatal intensive care in a Karen refugee camp: a 4 year descriptive study. *PLoS One*. 2013;8(8):e72721.

18. Thielemans L, Trip-Hoving M, Landier J, et al. Indirect neonatal hyperbilirubinemia in hospitalized neonates on the Thai-Myanmar border: a review of neonatal medical records from 2009 to 2014. *BMC Pediatr*. 2018;18(1):190.

19. Mabogunje CA, Olaifa SM, Olusanya BO. Facility-based constraints to exchange transfusions for neonatal hyperbilirubinemia in resource-limited settings. *World J Clin Pediatr*. 2016;5(2):182-90.

Confidential: For Review Only

20. Sanpavat S. Exchange transfusion and its morbidity in ten-year period at King Chulalongkorn Hospital. *J Med Assoc Thai*. 2005;88(5):588-92.

21. Radmacher PG, Groves FD, Owa JA, et al. A modified Bilirubin-induced neurologic dysfunction (BIND-M) algorithm is useful in evaluating severity of jaundice in a resource-limited setting. *BMC Pediatr*. 2015;15:28.

22. Thielemans L, Gornsawun G, Hanboonkunupakarn B, et al. Diagnostic performances of the fluorescent spot test for G6PD deficiency in newborns along the Thailand-Myanmar border: A cohort study. *Wellcome Open Res*. 2018;3:1.

23. Ebbesen F, Andersson C, Verder H, et al. Extreme hyperbilirubinaemia in term and near-term infants in Denmark. *Acta Paediatr*. 2005;94(1):59-64.

24. Kuzniewicz MW, Wickremasinghe AC, Wu YW, et al. Incidence, etiology, and outcomes of hazardous hyperbilirubinemia in newborns. *Pediatrics*. 2014;134(3):504-9.

25. Rijken MJ, Mulder EJ, Papageorghiou AT, et al. Quality of ultrasound biometry obtained by local health workers in a refugee camp on the Thai-Burmese border. *Ultrasound Obstet Gynecol*. 2012;40(2):151-7.

26. Moore KA, Simpson JA, Thomas KH, et al. Estimating Gestational Age in Late Presenters to Antenatal Care in a Resource-Limited Setting on the Thai-Myanmar Border. *PLoS One*. 2015;10(6):e0131025.

27. Barnett AL, Guzzetta A, Mercuri E, et al. Can the Griffiths scales predict neuromotor and perceptual-motor impairment in term infants with neonatal encephalopathy? *Arch Dis Child*. 2004;89(7):637-43.

28. Adoh TO, Woodhouse JM, Oduwaiye KA. The Cardiff Test: a new visual acuity test for toddlers and children with intellectual impairment. A preliminary report. *Optom Vis Sci.* 1992;69(6):427-32.

Confidential: For Review Only

29. Janet S, Carrara VI, Simpson JA, et al. Early neonatal mortality and neurological outcomes of neonatal resuscitation in a resource-limited setting on the Thailand-Myanmar border: A descriptive study. *PLoS One*. 2018;13(1):e0190419.

30. Haataja L, McGready R, Arunjerdja R, et al. A new approach for neurological evaluation of infants in resource-poor settings. *Annals of tropical paediatrics*. 2002;22(4):355-68.

31. Arnolda G, Nwe HM, Trevisanuto D, et al. Risk factors for acute bilirubin encephalopathy on admission to two Myanmar national paediatric hospitals. *Matern Health Neonatol Perinatol*. 2015;1:22.

32. Salas AA, Mazzi E. Exchange transfusion in infants with extreme hyperbilirubinemia: an experience from a developing country. *Acta Paediatr*. 2008;97(6):754-8.

33. Olusanya BO, Ogunlesi TA, Kumar P, et al. Management of late-preterm and term infants with hyperbilirubinaemia in resource-constrained settings. *BMC Pediatr*. 2015;15:39.

34. Shapiro SM, Popelka GR. Auditory impairment in infants at risk for bilirubin-induced neurologic dysfunction. *Semin Perinatol*. 2011;35(3):162-70.

35. Ip S, Chung M, Kulig J, et al. An evidence-based review of important issues concerning neonatal hyperbilirubinemia. *Pediatrics*. 2004;114(1):e130-53.

36. Boel M, Carrara VI, Rijken M, et al. Complex Interactions between soil-transmitted helminths and malaria in pregnant women on the Thai-Burmese border. *PLoS Negl Trop Dis.* 2010;4(11):e887.

37. Kemmer TM, Bovill ME, Kongsomboon W, et al. Iron deficiency is unacceptably high in refugee children from Burma. *J Nutr*. 2003;133(12):4143-9.

38. Swe ZY, Narksawat K, Chansatitporn N. Dental caries status and oral health behavior among Myanmar migrant workers in Mae Sot district, Tak Province, Thailand. *Myanmar*

Confidential: For Review Only

Dental Journal. 2016. http://www.mda-myanmar.org/wp-content/uploads/2015/01/Dental-Caries-Status-and-Oral-health-behaviour.pdf (Accessed 30 December 2019).

39. Turner C, Turner P, Carrara V, et al. High rates of pneumonia in children under two years of age in a South East Asian refugee population. *PLoS One*. 2013;8(1):e54026.

40. UNICEF. Situation analysis of children with disabilities in the Republic of the Union of Myanmar. Yangon: UNICEF, Myanmar; 2016. https://reliefweb.int/sites/reliefweb.int/files/resources/Full_report_in_English_0.pdf. (Accessed 30 December 2019).

Confidential: For Review Only

Table 1: Characteristics of neonatal period of 129 neonates with extreme NH

Characteristics		Extreme NH (n=129)
Year of admission	2009	22 (17.0%)
	2010	26 (20.2%)
	2011	12 (9.3%)
	2012	24 (18.6%)
	2013	27 (20.9%)
	2014	18 (14.0%)
Status	Refugee	91 (70.5%)
	Migrant	38 (29.5%)
Gender (male)		75 (58.1%)
Gestational age[a] ≥ 35 weeks		92 (71.3%)
Place of delivery	SMRU clinic	110 (85.3%)
	Tertiary hospital	4 (3.1%)
	Home	15 (11.6%)
Birth weight[b] (kg) (n=125) (median, IQR)		2.53 (1.96 – 2.90)
Small for Gestational Age[c] (n=125)		23 (18.4%)
Poor start to life[d]		15 (11.6%)
Mother primigravid		60 (46.5%)
Normal vaginal delivery		114 (88.4%)
Age at admission (days) (median, IQR)		1 (0 – 4)
Duration of admission (days) (median, IQR)		6 (4 – 11)
Extreme NH category[e]	Two SBR above ET	101 (78.3%)
	Rapid SBR rise + ABE signs	27 (20.9%)
	Clinical diagnosis of ABE	1 (0.8%)
Duration of phototherapy (hours) (median, IQR)		76 (48 – 124)
Peak SBR (µmol/L) (median, IQR)		430 (371 – 487)
Age at peak SBR (hours) (median, IQR)		97 (68 – 136)
Peak SBR at <72 hours of life		41 (31.8%)
Received exchange transfusion		4 (3.1%)
Potential ABO incompatibility[f] (n=126)		24 (19.0%)
G6PD-deficient male[g] (n=75)		33 (44.0%)
Polycythaemia[h]		10 (7.8%)
Birth trauma (visible bruising after birth recorded in the chart)		6 (4.7%)
Clinical diagnosis of sepsis[j] prior to onset of NH		34 (26.4%)

Data shown in number and (percentage) unless stated otherwise.

[a]Gestational age: Based on first trimester ultrasound.[(25)]

[b]Birthweight: weight measurement considered valid if done in the first 72 hours of life.

[c]Small for Gestational Age: Defined as birth weight below the 10th percentile for gestational age and sex, calculated with the Intergrowth-21st newborn size application tool (https://intergrowth21.tghn.org).

[d]Poor start to life: Apgar score <7 at 5 minutes and/or clinical suspicion of meconium aspiration and/or resuscitation at birth with at least five inflation breaths.

Confidential: For Review Only

[e]Extreme NH categories: i) two consecutive serum bilirubin (SBR) measurements above the exchange transfusion (ET) threshold of the NICE guideline; ii) SBR levels rising faster than 8.5 µmol/L per hour in combination with one SBR measurement above the ET threshold or with clinical symptoms of acute bilirubin encepalopathy (ABE); iii) a clinical diagnosis of ABE.
[f]Potential ABO incompatibility: Mother-fetus pairs with mother blood group “O” and neonate blood group “A” or “B”. Rhesus factor and Coombs test were not available.
[g]Diagnosed by G6PD fluorescent test.[(22)]
[h]Polycythaemia: Two consecutive haematocrit values >70% from a capillary sample, or a diagnosis of polycythaemia recorded in the clinical chart.
[i]Clinical diagnosis of sepsis: sepsis reported as clinical diagnosis treated at least 5 days by intravenous antibiotics (blood culture confirmations were not available).

Confidential: For Review Only

Table 2: Neonatal and maternal characteristics of extreme NH survivors present for neurodevelopmental assessment (n=37) and those unavailable or untraceable (n=78)

Characteristics	Present (n=37)	Untraceable (n=78)	p-value
Gender (male)	20 (54.1%)	47 (60.3%)	0.529
Year of birth (2012-2014)	24 (64.9%)	41 (52.6%)	0.214
Refugee status	23 (62.1%)	54 (69.2%)	0.452
First born	20 (54.1%)	44 (56.4%)	0.812
EGA (weeks + days) (median, IQR)	37+4 (34+5 – 38+3)	37+4 (35+1 – 38+6)	0.578
EGA <35 weeks	11 (29.7%)	18 (23.1%)	0.443
Small for gestational age	5/35 (14.3%)	14/75 (18.7%)	0.571
Normal vaginal delivery	31 (83.8%)	70 (89.7%)	0.361
Days in SCBU (median, IQR)	8 (5 – 16)	7 (4 – 13)	0.201
Clinical diagnosis of sepsis prior to onset of NH	8 (21.6%)	18 (23.1%)	0.862
Maternal age (median, IQR)	24 (20 – 30)	22 (19 – 28)	0.391
Maternal illiteracy	9/27 (33.3%)	23/57 (40.4%)	0.536
Maternal smoking	7 (18.9%)	11 (14.3%)	0.525

Data shown in number and (percentage) unless stated otherwise.

Confidential: For Review Only

Table 3. Long term clinical, visual, neurological, and neurodevelopmental outcomes of 37 survivors of extreme NH by gestational age category

	Gestational Age <35 weeks (n=11)	Gestational Age >=35 weeks (n=26)	All (n=37)
**General characteristics**			
Age at testing (months) (median, IQR)	62.5 (36.5 – 93.0)	43.8 (34 – 64.5)	51.0 (35 – 81)
Age < 4 years old	3 (27.3%)	13 (50.0%)	16 (43.2%)
Gender (male)	4 (36.4%)	16 (61.5%)	20 (54.1%)
First born	8 (72.7%)	12 (46.2%)	20 (54.1%)
Received Exchange Transfusion	0	3 (3.1%)	3 (8.1%)
Refugee status	7 (63.6%)	16 (61.5%)	23 (62.2%)
**Clinical outcome**			
Being Unwell[a]	3 (27.3%)	8/25 (32.0%)	11/36 (30.6%)
Stunting[b]	2 (18.2%)	13 (50.0%)	15 (40.5%)
Teeth abnormality[c]	5/10 (50.0%)	13/26 (50.0%)	18/36 (50.0%)
**Visual and neurological outcomes**			
Visual examination abnormality[d]	1 (9.1%)	7 (26.9%)	8 (21.6%)
Neurological exam abnormality[e]	0	2 (7.7%)	2 (5.4%)
**Neurodevelopmental outcome**			
Maternal perception of child's development: mother has worries	3 (27.3%)	3 (11.5%)	6 (16.2%)
**GMDS scores**			
General percentile score (median, IQR)	32 (11 – 42)	7 (<1 – 42)	11 (2 – 42)
Poor development (<10th centile)	2 (18.2%)	14 (53.8%)	16 (43.2%)
Delayed development (10-49th centile)	7 (63.6%)	10 (38.5%)	17 (46.0%)
Normal development (≥50th centile)	2 (18.2%)	2 (7.7%)	4 (10.8%)
Locomotion (median, IQR)	34 (19 – 59)	12 (3 – 58)	19 (5 – 58)
Personal and social (median, IQR)	23 (8 – 94)	32 (15 – 64)	26 (11 – 64)
Hearing and language (median, IQR)	12 (6 – 57)	11 (<1 – 33)	11 (<1 – 33)
Eye and hand co-ordination (median, IQR)	27 (4 – 46)	14 (4 – 48)	22 (4 – 46)
Performance (median, IQR)	26 (6 – 80)	3 (<1 – 19)	4 (<1 – 40)
Practical reasoning (median, IQR)[f]	18 (2 – 51)	11 (1 – 43)	14 (2 – 49)

Data shown in number, and (percentage).
[a]Being Unwell: more than one hospitalization per year or presence of a chronic disease as reported in the child's medical booklet.
[b]Stunting: height-for-age z-scores below -2 as calculated with WHO Child Growth Standards (http://www.who.int/childgrowth/en/).
[c]Teeth abnormality: judgement of medical staff looking for the presence of black staining or cavities, confirmed by first author through photographic records.

Confidential: For Review Only

[d]Visual examination abnormality: unconjugated eye movement and Cardiff's binocular visual contrast and visual acuity lower than the normal values by age categories (instructions manual for the Cardiff Acuity Test).

[e]Neurological examination abnormality: judgement of the medical staff after general observation while moving, and evaluating tone, strength and sensibility, coordination, gait and posture, and reflexes.

[f]One child had an adjusted age < 2 years old when tested, therefore practical reasoning was only available for 10 preterm children.

Confidential: For Review Only

422 **Figures**

423 **Figure 1: Location of the three Shoklo Malaria Research Unit (SMRU) clinics.**

424 The three clinics are: Mae La clinic for refugees; Wang Pha and Maw Ker Thai clinics for
425 migrant population. SMRU main office is located in Mae Sot town.

426 Photo credit: Daniel Parker.

427

428

429 **Figure 2: Flowchart representing the selection of neonates with extreme**
430 **hyperbilirubinemia (NH).**

431 [a]Major congenital abnormality was defined as an abnormal finding on surface examination
432 and/or auscultation of the heart and lungs reported in the medical chart and confirmed by a
433 medical doctor.

434

Confidential: For Review Only

Competing interests

The authors have declared that no competing interests exist.

Patient and Public Involvement

The Tak Province Border Community Ethics Advisory Board members representing the local community where the study took place reviewed the protocol and their input into the design of the project was taken into account.

Funding

Shoklo Malaria Research Unit is part of the Mahidol-Oxford University Tropical Medicine Research Program at the Faculty of Tropical Medicine, Mahidol University, Bangkok, Thailand, and supported by the Wellcome Trust of Great Britain. The funders had no role in study design, data collection and analysis, decision to publish, or preparation of the manuscript. LT was supported by a PhD grant from 'The Belgian Kids' Fund for Pediatric Research.

Contribution

EMNW, LT, VIC conceived and designed the study, and BH, PFR, CT, FHN and RM contributed to the study design. EMNW, LT and VIC coordinated the study and supervised the research teams. MCD, AAN, WS, SK performed all the neurodevelopment tests and clinical examinations. TN developed the data collection and quality controlled. EMNW, LT and VIC led the analysis and interpretation of the data with JL leading statistical analysis. All authors supported the preparation and later critically reviewed the manuscript including in the final approval of the manuscript. All authors had full access to all of the data (including statistical

Confidential: For Review Only

459 reports and tables) in the study and can take responsibility for the integrity of the data and

460 the accuracy of the data analysis.

Confidential: For Review Only

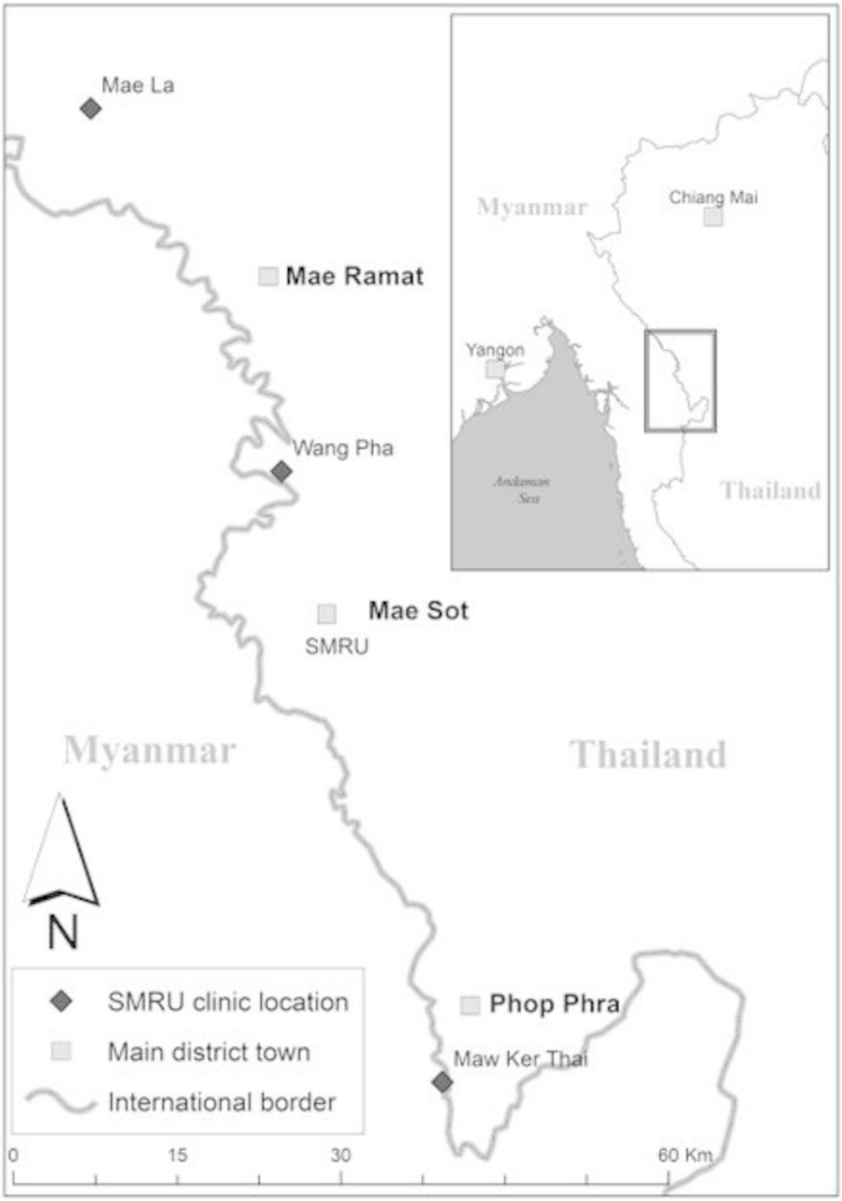

115x163mm (300 x 300 DPI)

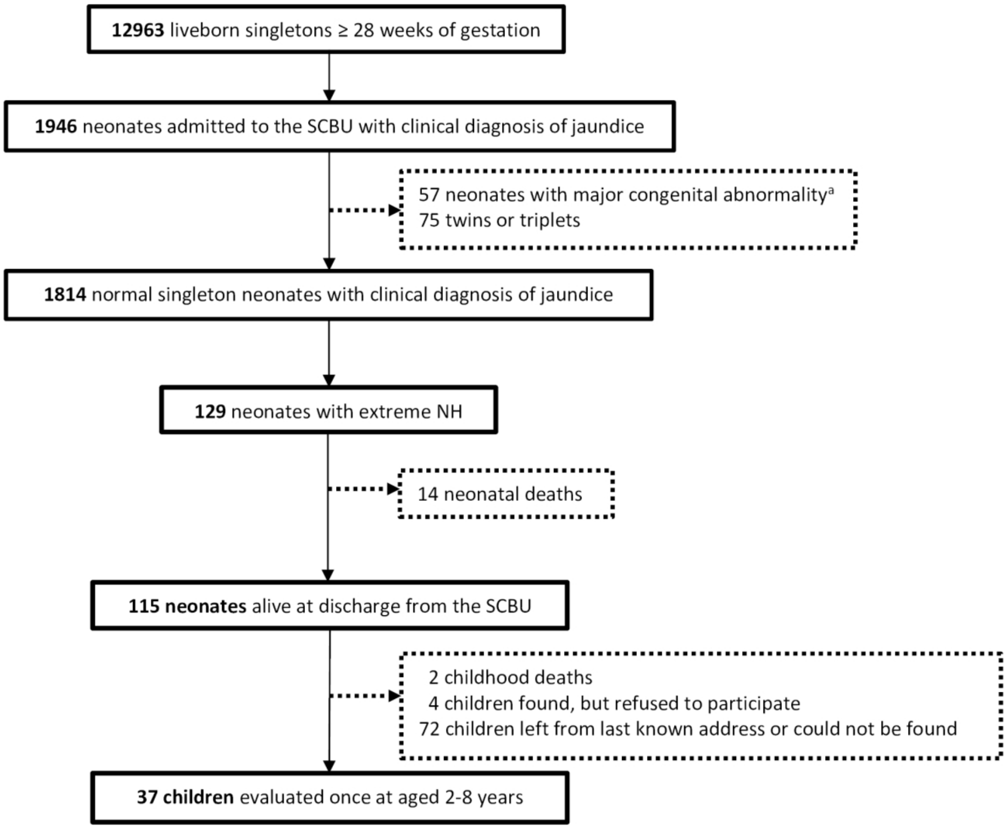

166x136mm (300 x 300 DPI)
